# In-Depth Immunological Typization of Children with Sickle Cell Disease: A Preliminary Insight into Its Plausible Correlation with Clinical Course and Hydroxyurea Therapy

**DOI:** 10.3390/jcm11113037

**Published:** 2022-05-27

**Authors:** Giulia Giulietti, Daniele Zama, Francesca Conti, Mattia Moratti, Maria Teresa Presutti, Tamara Belotti, Maria Elena Cantarini, Elena Facchini, Mirna Bassi, Paola Selva, Elisabetta Magrini, Marcello Lanari, Andrea Pession

**Affiliations:** 1 Specialty School of Paediatrics, University of Bologna, 40138 Bologna, Italy; giulia.giulietti@hotmail.it (G.G.); mattia.moratti@studio.unibo.it (M.M.); 2 Pediatric Emergency Unit, IRCCS Azienda Ospedaliero-Universitaria di Bologna, 40138 Bologna, Italy; daniele.zama2@unibo.it (D.Z.); marcello.lanari@unibo.it (M.L.); 3 Pediatric Unit, IRCCS Azienda Ospedaliero-Universitaria di Bologna, 40138 Bologna, Italy; andrea.pession@unibo.it; 4 Otolaryngology Department, University Hospital of Modena, 41125 Modena, Italy; mtpresutti@gmail.com; 5 Pediatric Oncology and Hematology Unit “Lalla Seràgnoli”, IRCCS Azienda Ospedaliero-Universitaria di Bologna, 40138 Bologna, Italy; tamara.belotti2@unibo.it (T.B.); mariaelena.cantarini@aosp.bo.it (M.E.C.); elena.facchini@aosp.bo.it (E.F.); mirna.bassi@ausl.bo.it (M.B.); 6 Laboratory of Immuno-Haematology-Laboratorio Unico Metropolitano, Azienda USL, 40133 Bologna, Italy; paola.selva@ausl.bo.it (P.S.); elisabetta.magrini@ausl.bologna.it (E.M.)

**Keywords:** children, clinical course, hydroxyurea, immunological typization, immunophenotyping, pediatric, sickle cell disease

## Abstract

Sickle cell disease (SCD) is a condition of functional hypo-/a-splenism in which predisposition to bacterial infections is only a facet of a wide spectrum of immune-dysregulation disorders forming the clinical expression of a peculiar immunophenotype. The objective of this study was to perform an in-depth immunophenotypical characterization of SCD pediatric patients, looking for plausible correlations between immunological biomarkers, the impact of hydroxyurea (HU) treatment and clinical course. This was an observational case–control study including 43 patients. The cohort was divided into two main groups, SCD subjects (19/43) and controls (24/43), differing in the presence/absence of an SCD diagnosis. The SCD group was split up into HU+ (12/19) and HU− (7/19) subgroups, respectively receiving or not a concomitant HU treatment. The principal outcomes measured were differences in the immunophenotyping between SCD patients and controls through chi-squared tests, t-tests, and Pearson’s correlation analysis between clinical and immunological parameters. Leukocyte and neutrophil increase, T-cell depletion with prevalence of memory T-cell compartment, NK and B-naïve subset elevation with memory and CD21low B subset reduction, and IgG expansion, significantly distinguished the SCD HU− subgroup from controls, with naïve T cells, switched-memory B cells and IgG maintaining differences between the SCD HU+ group and controls (*p*-value of <0.05). The mean CD4+ central-memory T-cell% count was the single independent variable showing a positive correlation with vaso-occlusive crisis score in the SCD group (Pearson’s R = 0.039). We report preliminary data assessing plausible clinical implications of baseline and HU-related SCD immunophenotypical alterations, which need to be validated in larger samples, but potentially affecting hypo-/a-splenism immuno-chemoprophylactic recommendations.

## 1. Introduction

Sickle cell disease (SCD) is an inherited hemoglobin disorder characterized by a pro-inflammatory state, associated with an early loss of splenic function in terms of senescent/dysfunctional erythrocyte filtration and of pathogen clearance through mechanisms involving both the innate and adaptive immune systems. One of the major issues is thus represented by infectious susceptibility, especially towards encapsulated bacteria, such as Streptococcus pneumoniae, Neisseria meningitidis and Haemophilus influenzae [1,2,3,4,5]. Life-threatening overwhelming post-splenectomy infections, sustained by the above-mentioned bacterial pathogens, can potentially affect all individuals with splenic dysfunction, which can be post-surgical, congenital or functional [6,7]. However, predisposition to bacterial infections is only a facet of a wide spectrum of immune-dysregulation disorders, including autoimmune, allergic, autoinflammatory and vascular complications, which can develop as typical manifestations of a so-called condition of functional hypo-/a-splenism [3].

Regarding infective prevention, to date, there is no consensus in worldwide guidelines on the type, optimal duration and target population of prophylactic treatments specific for the three main categories of hypo-/a-splenism [5,7].

As concerns SCD management, hydroxyurea (HU) is a safe primary disease-modifying therapy offered to affected infants from 9 months of age regardless of clinical severity, according to National Heart, Lung and Blood Institute guidelines drawn from the multicentric BABY HUG study, showing an HU-related long-term reduction in vaso-occlusive crises along with improved growth and spleen function, evidenced via liver–spleen scintigraphy [8,9,10,11,12].

HU exerts its therapeutical role in the promotion of antioxidant gene expression, dampening inflammation susceptibility, in the reduction of the pro-inflammatory-cytokine-producing classical monocyte subset in favor of the patrolling subset, supporting a virtuous circulating rheology, and in the inhibition of the ribonucleotide reductase activity, reducing leukocytic count and impairing the maturational process of T-cell compartment from naïve to terminally differentiated clones [13,14].

Moreover, few studies in the literature have taken into account the SCD patients’ immunophenotypical characterization and the potential impact of specific treatments on the immune system, with a total lack of pediatric study analyzing both humoral and cellular immunity of SCD-related hypo-/a-splenic individuals [15,16,17,18,19].

Even fewer studies have looked into the immunological effects of HU in children with SCD, without, however, assessing the correlation between SCD clinical course and HU-related immunophenotypical alterations [20,21].

Based on the aforementioned considerations, we conducted a study focused on a wide and in-depth immunophenotype characterization of SCD patients, taking into account distinctly each T-/B-cell compartment subset.

We further looked for plausible correlations existing between specific immunological biomarkers, the impact of treatments and clinical course, especially in terms of vaso-occlusive crises and infectious susceptibility.

We subsequently searched for reasonable correlations existing between HU-related immunological asset alterations and treatment effectiveness in terms of vaso-occlusive crises and infectious susceptibility.

## 2. Materials and Methods

This observational case–control study included 43 patients attending the outpatient clinic of the Hematology and Immunology Service of the Pediatric Unit, IRCCS, Azienda Ospedaliero-Universitaria di Bologna, between January 2018 and July 2020. The cohort was divided into two main groups, SCD subjects and controls, differing in the presence/absence of previously diagnosed SCD.

### 2.1. SCD Group Inclusion Criteria

Inclusion criteria for the SCD group were the following: diagnosis of SCD ascertained by standard clinical–laboratoristic procedures [1,3]; age 0–18 years at the time of SCD diagnosis; and enrollment contingent on the previous ascertainment of the absence of underlying inborn errors of immunity and secondary immunodeficiencies due to non-hematological disorders, apart from SCD-related functional hypo-/a-splenism.

### 2.2. SCD Group Characterization

The SCD group numbered 19 patients (44%), 9 females (47%) and 10 males (53%), with a mean age of 11 years (3–23 years).

As regards ethnicity, 14 subjects were African (74%) and 5 were Caucasian (26%).

The hemoglobin genotype distribution was the following: 12/19 (63%) HbSS, 6/19 (32%) HbSβ0-thalassemic and 1/19 (5%) HbSC. The SCD group was further split up into SCD HU+ and SCD HU− subgroups, respectively receiving or not concomitant treatment with HU. The SCD HU+ subgroup consisted of 12/19 individuals (63%) treated at a median dose of 21 mg/kg/die (15–30 mg/kg/die). The mean age of onset of therapy was 10 years (2–18 years) and the mean duration of therapy was 4 years (2–13 years). Additional monthly erythrocyte exchange was necessary only for a single patient of this subgroup (8%). Further details about the SCD group are reported in Table 1.

### 2.3. Control Group Inclusion Criteria

Controls were recruited from among patients referred to the Hematology and Immunology Service of the Pediatric Unit, IRCCS, Azienda Ospedaliero-Universitaria di Bologna, by reason of recurrent infections, reactive lymphadenopathy and abdominal pain—all common causes of referral to a pediatric ward.

The entire control group underwent an in-depth immunological characterization seldom applied in a strictly hematologic context, due to the close cooperation within the Hematology and Immunology Pediatric Service, showing normal distribution, differentiation and development of T-cell and B-cell subsets linked to para-physiological common recurrent phlogosis of childhood not requiring therapy.

The inclusion criteria for the control group were the following: an apparent base health condition with an AA hemoglobin genotype; enrollment contingent on the previous assessment of normal clinical–laboratoristic examinations; and age- and sex-matching with the SCD group.

### 2.4. Control Group Characterization

The control group included 24 age- and sex-matched subjects, 11 females (46%) and 13 males (54%), in an apparent base health condition with an AA hemoglobin genotype. A more detailed characterization of the controls is reported in Table 2.

We executed a systematic comparison between the SCD group and controls, the SCD HU− subgroup and controls, the SCD HU+ subgroup and controls, and the SCD HU+ and SCD HU− subgroups through an exhaustive, comprehensive report of clinical and laboratoristic data.

All patients and their legal guardians provided written informed consent to participate in this study, carried out in accordance with the Declaration of Helsinki. Recruitment was performed in the Onco-hematology Unit and Immunology Clinic during regular medical check-ups according to clinical practice. Written informed consent and agreement to disclosure of information in publications was obtained from patients or their legal guardians/next of kin, with the option to withdraw from the study at any moment by communicating their decision to us. The biological samples were obtained from patients’ peripheral blood exclusively on the occasion of diagnostic procedures after informed consent, without exposing patients to further check-up visits and blood sampling finalized exclusively for research.

### 2.5. Clinical Course of the SCD Group

The main items considered to describe the SCD group were the following: infection score, defined as the mean number of infections per year over a minimum period of three years; crisis score, defined as the mean number of vaso-occlusive painful events per year over a minimum period of three years; type of painful crises (priapism, cerebrovascular accident, acute chest syndrome, dactylitis, bone infarcts); age at the beginning of HU therapy; HU dosage; and eventual previous splenectomy.

### 2.6. Laboratory Investigations

Laboratory investigations were oriented to the characterization of immunological assets, including: leukocyte formula (Sysmex XN-20) [22]; extended lymphocyte immunophenotyping [CD3+ (PAN-T), CD3+CD4+ (helper), CD4+CD45RA+CCR7+ (naїve), CD4+CD45RA−CCR7+ (central memory), CD4+CD45RA−CCR7− (effector memory), CD4+CD45RA+CCR7− (terminal effector memory), CD3−CD8+ (cytotoxic), CD8+CD45RA+CCR7+ (naїve), CD8+CD45RA−CCR7+ (central memory), CD8+CD45RA−CCR7− (effector memory), CD8+CD45RA+CCR7− (late effector memory), CD4+CD127−CCR7+CD25++ (regulatory) (T-reg) and TCRαβ+CD3+CD4−CD8− double-negative T cells, CD4+/CD8+ ratio, CD3+γ+δ+ cells, CD56+CD16+CD3− (natural killer) NK cells, CD19+ (PAN-B), CD19+IgD+CD27− (naїve), CD19+IgD+CD27+ (memory), CD19+IgD−CD27+ (switched-memory), CD19+IgM++CD38++ (transitional), CD19+CD21+lCD38− (CD21low) and CD19+IgM−+CD38++ (plasmablast) B cells] through multiparametric flow cytometry [23]; and serum immunoglobulin levels (IgG and subclasses, IgA, IgM and IgE) through an immunoturbidimetric method (Beckman Coulter, Brea, CA, USA) [24].

In order to execute lymphocyte typization, whole blood on EDTA was stained with an appropriate antibody cocktail (Becton Dickinson, Franklin Lakes, NJ, USA) for 30 min at room temperature. Afterward, red blood was supplemented with 0.5 mL 1 × BD FACS Lysing solution, washed with PBS and suspended in PBS. Samples were acquired on a Facs Canto II (BD Bioscience, San Diego, CA, USA) and analyzed using Diva software; the analysis of each patient typically included between 10,000 and 50,000 PBMCs, depending on sample availability.

In order to avoid any possible alteration linked to immune system activation and to maintain the same setting of immunophenotyping execution in the SCD and control groups, immunological data were age-referenced and gathered in the absence of clinical–laboratoristic evidence of infections at the time of blood sampling, at a distance of at least three months from vaccine administrations and of at least six months from blood transfusions, except for a single patient undergoing monthly erythrocyte exchange.

Nevertheless, this fact did not affect statistical analysis, as the immunophenotype detection executed before the start of erythrocyte exchange showed similar results to that performed subsequently to the procedure.

Of the 19 patients, the 7 in the SCD HU+ group were on prophylactic amoxicillin therapy at the time of immunological evaluation, while none of the controls were receiving antibiotic therapy.

At first glance, this could be considered a bias. In the literature, there are studies reporting amoxicillin’s effects on the human immune system in terms of functional impairment of polymorphonuclear leukocytes, altering their chemotaxis and migration to inflammation sites; however, there have been no reported effects of amoxicillin in terms of impaired numerical distribution and differentiation of the various innate and adaptive cell subsets circulating in blood.

### 2.7. Statistical Analysis

Descriptive statistics included the mean (95% confidence interval) as appropriate for continuous variables and the frequency for categorical variables. All analyses were performed using STATA software version 7.0 and Microsoft Excel version 2013.

### 2.8. Statistical Methods

Data elaboration, based on the comparison between different groups, aimed to assess the statistical significance (two-tailed *p*-value of <0.05) of a difference emerging from the groups through chi-squared tests for frequency and Student’s t-test for the mean, and to describe the correlation (two-tailed *p*-value of <0.05) between variables within a group through the Pearson correlation coefficient.

The relationship between infection score per year and the continuous variables examined was evaluated at the same time-point by univariate and multivariate analysis of correlations for the whole population.

Multivariable analysis with linear regression was performed to control for potential confounding variables of age, sex, and history of splenectomy.

## 3. Results

### 3.1. SCD HU− Subgroup

Immunophenotyping revealed a leukocyte and neutrophil increase, a T-cell depletion with a prevalence of memory T-cell compartment, NK and B-naïve subset elevation with memory and CD21low B subset diminishment, and IgG expansion in the SCD HU− subgroup compared to controls.

As shown in Table 3, statistically significant differences emerging from the two groups regarded the mean leukocyte and neutrophil absolute count; CD3+ (PAN-T); CD4+ helper, naïve, central memory and effector memory T-cell%; NK-cell%; CD19+ naïve, memory, switched-memory, and CD21low B-cell%; and serum IgG levels.

As concerns the leukocytic formula, the mean leukocyte absolute count was superior (*p* = 0.001) in the SCD HU− subgroup, as was the mean neutrophil absolute count (*p* = 0.016).

Considering T-cell subsets, CD3+ (PAN-T) cell% was, on average, inferior (*p* = 0.000) in the SCD HU− subgroup, and the same goes for CD4+ helper T-cell% (*p* = 0.032) and CD4+ naïve T-cell% (*p* = 0.021). CD4+ central memory T-cell% was, on average, superior (*p* = 0.002) in the SCD HU− subgroup, and the same applies to effector memory T-cell% (*p* = 0.039).

As regards the NK-cell%, their mean count was higher (*p* = 0.044) in the SCD HU− subgroup.

As for B-cell subsets, the CD19+ naïve B-cell% was, on average, higher (*p* = 0.001) in the SCD HU− subgroup, while the CD19+ memory B-cell% was, on average, lower (*p* = 0.000) in the SCD HU− subgroup, as were the CD19+ switched-memory B-cell% (*p* = 0.009) and CD21low B-cell% (*p* = 0.029).

Finally, the mean IgG count was superior (*p* = 0.001) in the SCD HU− subgroup. 

The difference in mean CD4+ central memory T-cell% and memory B-cell% was statistically significant between the SCD HU− subgroup and controls.

### 3.2. SCD HU+ Subgroup

Immunophenotyping revealed naïve T-cell and switched-memory B-cell depletion along with IgG and IgA elevation in the SCD HU+ subgroup compared to controls.

As shown in Table 3, the statistically significant differences in immunological parameters between the two groups involved mean CD4+ naïve T-cell%, switched-memory B-cell% and serum IgG and IgA count. In particular, the CD4+ naïve T-cell% was, on average, inferior in the SCD HU+ subgroup (*p* = 0.045); the same goes for the switched-memory B-cell% (*p* = 0.014).

On the contrary, the mean IgG level was superior in the SCD HU+ subgroup (*p* = 0.000); the same applies to the mean IgA level (*p* = 0.000).

### 3.3. SCD HU− vs. SCD HU+ Subgroups

Immunophenotyping revealed leukocyte and CD4+ effector memory T-cell expansion in contrast with PAN-T cell shrinkage in the SCD HU− subgroup compared to the SCD HU+ subgroup.

The difference in mean CD4+ central memory T-cell% and memory B cells lost its significance in the SCD HU+ subgroup when compared to controls.

**Figure 1:**
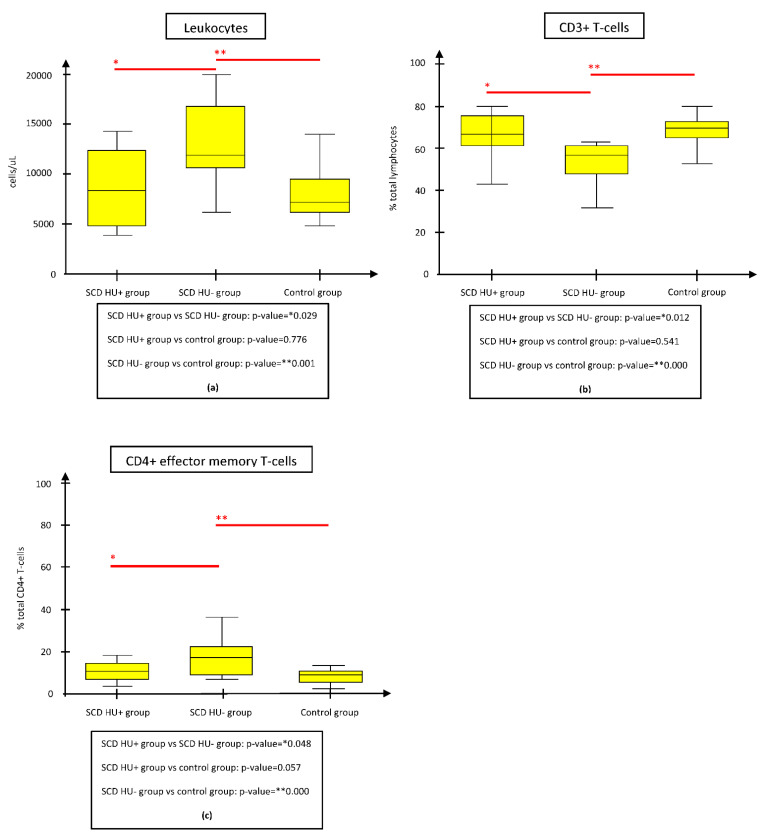
Comparison of immunologic parameters between the SCD HU− and HU+ groups, the SCD HU− and control groups, and the SCD HU+ and control groups. The figure shows only the parameters whose difference resulted statistically significant both between SCD HU- and HU+ groups and between SCD HU- and control groups. (**a**) Mean WBC absolute count was significantly reduced in SCD HU+ subgroup and controls respect to SCD HU- subgroup. (**b**) Mean CD3+ T-cell% count was significantly augmented in SCD HU+ subgroup and controls respect to SCD HU- subgroup. (**c**) Mean CD4+ effector memory T-cell% count was significantly lower in SCD HU+ subgroup and controls respect to SCD HU- subgroup. Abbreviations: HU, hydroxyurea; SCD, Sickle cell disease.

As reported in Figure 1a,c, the only statistically significant differences regarding the two groups appeared for the absolute leukocytes (*p* = 0.029) and CD4+ effector memory T-cell% (*p* = 0.048), on average more represented in the SCD HU− subgroup, along with mean CD3+ (PAN-T) cell% (*p* = 0.012), less represented in the SCD HU− subgroup.

### 3.4. SCD Group

Correlation analysis shed light on the clinical implications of baseline and HU-related immunophenotypical alterations.

In univariate analysis, we observed a statistically significant negative correlation between mean memory B-cell% count and the abovementioned crisis score in the SCD group (Pearson’s R = 0.010) (Supplementary Table S1).

In multivariate analysis, mean CD4+ central memory T-cell% count was the single independent variable showing a statistically significant positive correlation with crisis score in the SCD group (Pearson’s R = 0.039, Standard Regression Coefficient ß = 0.89) (Supplementary Table S1).

Each of the differences between the SCD and control groups detected through univariate analysis retained statistical significance when controlling for sex, age and previous splenectomy as confounding variables.

## 4. Discussion

To date, in the literature, there is a lack of a complete immunophenotypical characterization of SCD subjects also taking into account T-cell and B-cell compartments, especially in a pediatric setting. Our study contributes to a wide and in-depth characterization of the immunological profile of SCD patients, taking into account distinctly each T-/B-cell compartment’s subset.

Moreover, HU-induced alterations in immunological assets in SCD children have been investigated so far only by Lederman et al. [20] and Nickel et al. [21], with important limitations.

While the former’s analysis was restricted only to the T-cell compartment and IgG response to pneumococcal and MMR vaccines [20], the latter’s study also examined innate immune system cells (leukocytes, neutrophils, monocytes, NK cells), but failed to include immunoglobulins and B-cell subsets, except for naïve and memory B cells [21].

The key strength of our project relies in the performing of an in-depth immunological analysis on SCD in a pediatric context, comprehensive of all T- and (almost) all B-cell subsets, including also IgG, IgA, IgM and IgE, in an attempt to attribute clinical implications both to SCD baseline and to HU-related immunological parameters.

Furthermore, our study is the first to separately analyze the two CD19+ memory B-cell components (memory and switched-memory) in an SCD pediatric population, including also transitional, CD21low and plasmablastic B-cell subsets. In the literature, only one previous study, conducted on adult SCD subjects, distinguished these two B-cell subpopulations in the immunophenotypical assessment [25].

Through systematic comparisons and correlation analysis, we highlighted how HU therapy tends to bring back SCD immunological assets to a base health condition. Thus, the changes in these parameters could theoretically serve as indicators of HU effectiveness in terms of clinical course and spleen functioning, eventually helping in therapeutic dose adjustment and immuno-chemoprophylactic recommendations.

The SCD HU− subgroup showed significantly higher mean leukocyte and neutrophil absolute count and mean CD4+ central memory and effector memory T-cell% count in comparison to controls, while CD3+ (PAN-T) cell% and CD4+ helper T-cell% were, on average, inferior when compared to controls. These results have a counterpart in the literature, where immunophenotyping of SCD patients off therapy was characterized by neutrophilic, lymphocytic and monocytic leukocytosis, along with a prevalence of memory on naïve T cells, interesting in a particular CD8+ subset in a context of T-cell compartment enlargement [5,15,16,17,18,19,20,21]. However, other immunological features typical of SCD, such as expansions in CD8+ memory T-cell% and CD19+ B-cell% subsets and inversion of the CD4+/CD8+ ratio, were not observed in the SCD HU− subgroup, probably due to the small sample size [5,20,21].

An issue worth discussing is the greater mean level of NK-cell% reported in the SCD HU− subgroup with respect to controls, fitting with the literature [21,26]. This population is thought to be distinguished into two components: a naïve subset, predominantly resident in lymphoid tissues, such as the spleen, mainly involved in cytokine production and responsible for virtuous innate/adaptive immunity cross-signaling, regulating CD4+ naïve differentiation into type 1, 2, and 17 helper T cells; and a memory subset, resident in peripheral blood, have a cytotoxic function and producing a huge quantity of IFN-γ [26,27].

Thus, SCD-related spleen dysfunction could affect the resident naïve NK-cell%, causing a compensative expansion of the circulating memory NK-cell%, detected by flow cytometry as NK cells and so resulting in a higher mean NK-cell% count in the SCD group.

The prevalence of memory on the naïve NK-cell% could be accounted for by two IFN-γ-regulated facts: the promotion of CD4+ and CD8+ naïve to memory differentiation by a persistent secondary adaptive immune response, typical of SCD-related immune-dysregulation [28]; and the suppression of CD4+ follicular type 1 helper T cells, leading to a delayed germinal center response with a subsequent predominance of naïve on memory B-cell% and a paucity of activated CD21low B-cell% [29].

As concerns B-cell compartment, the SCD HU− subgroup showed a superior mean CD19+ naïve B-cell% count together with an inferior mean of CD19+ memory, switched-memory and CD21low B-cell% when compared to controls. These findings may be due to the SCD-related functional hypo-/a-splenic state, characterized by the aforementioned IFN-γ-associated immune dysregulation [29].

Literature reports have indicated a significant increase in both absolute and percentage CD19+ total B-cell count in patients suffering from SCD not on HU as compared to healthy subjects, along with augmented CD19+ naïve B cells, matching in part with our results [18,20,21].

As regards serum immunoglobulins, our data are partially supported by the literature, as in the SCD HU− subgroup, IgG significantly expanded when compared to individuals with a base health condition; the same goes for IgA, although this finding did not reach statistical significance, probably as a consequence of the low sample size [30]. Moreover, IgM decline in the SCD HU− subgroup, albeit not reaching statistical significance, was potentially due to functional hypo-/a-splenism-related deficiency of antibody-producing (switched) memory B-cell subsets, corresponding to what has been recently observed by Cherif-Alami et al., although discordant findings emerged from previous studies in the literature [7,18,30,31,32]. Our results could be explained by the above-mentioned compensatory output of IFN-γ-producing memory NK cells, directly activating B-cell production of IgG and promoting IgM class switching to IgG and IgA [31,33,34].

Further, our study confirmed a higher mean IgE level among SCD HU− pediatric patients, consistent with an adaptive cellular immunity skewed towards a T-helper 2 phenotype; however, this was without statistical significance, ascribable to the small cohort size. An increase in IgE levels occurs much more frequently in children affected by SCD than in the general pediatric population, being associated with a higher risk for both asthma and acute chest syndrome, as a result of an imbalance between type 1 and 2 helper T cells, with adaptive cellular immunity dominated by the latter [35].

We found that the mean CD4+ central memory T-cell% count was the only independent variable showing a positive correlation with crisis score in the multivariate analysis. This association probably reflects the role of persistent/chronic immune activation sustained by SCD-related inflammatory and infectious susceptibility in the impairment of T-cell regulation of B cells, favoring the differentiation process from naïve to memory T cells and eventually resulting in an immunosenescent-like phenotype [5,19,29].

Interestingly, in the univariate analysis, a significant negative correlation between mean memory B-cell% count and crisis score also emerged. This may be ascribed to the fact that in functional hypo-/a-splenism, the impairment of senescent/dysfunctional erythrocyte filtration runs parallel with vicious T-cell/B-cell compartment cross-signaling, with a subsequent faulty differentiation of CD19+ naïve into memory and switched-memory B cells [2,4,5,18,32,36].

In the SCD HU+ subgroup, treated in a dosage range of 15–30 mg/kg/day, we observed immunophenotypical assets similar to those detected in the control group, with the exception of the CD4+ naïve T-cell% and CD19+ switched-memory B-cell%, being, on average, significantly lower in patients on HU therapy. This could be due to the fact that not all SCD HU+ subgroup members received an HU dose of at least 20 mg/kg/day, which was the minimum dosage used in the BABY-HUG study; furthermore, not all subjects reached the maximum tolerated HU dose, although more than two painful crises per year occurred in some of them. Eventually, it should be considered that the CD4+ naïve T-cell% and CD19+ switched-memory B-cell% diminishment could be intrinsic features of SCD-related functional hypo-/a-splenism, unmodifiable by HU [10,37,38,39]. This immunological resetting, being in correspondence with literature reports [20,21], is probably due to the dampening of the SCD-related chronic pro-inflammatory baseline condition.

Differences in the mean leukocyte count, CD3+ (PAN-T) cell% and CD4+ effector memory T-cell% were the only parameters maintaining statistical significance both between the SCD HU− and HU+ subgroups and between the SCD HU− subgroup and controls, but not between the SCD HU+ subgroup and controls (Figure 1a–c). This strengthens the hypothesis that leukocyte and CD4+ effector memory T-cell% expansion, along with CD3+ (PAN-T) cell% shrinkage, are constitutive features of SCD, potentially modulable by HU therapy. Leukocytosis mirrors the pro-inflammatory state linked to SCD-related chronic inflammation- and infection-driven immune activation [5].

CD3+ (PAN-T) cell% deficiency could be a sign of impaired T-cell neogenesis due to SCD-related thymic dysfunction, as well as the consequence of the above-mentioned augmented output of memory NK cells and CD19+ B cells as a compensatory mechanism supplying hypo-/a-splenism [26,29,40].

Eventually, CD4+ effector memory T cell production could be favored by a persistent secondary immune system response due to both infectious susceptibility and IFN-γ-related effects of memory NK cells, creating a pool of activated T-clones ready to take on effector functions [19]. While the CD4+ central memory T-cell% population was significantly expanded in the SCD HU− subgroup, it showed normal values in the SCD HU+ subgroup; thus, considering its aforementioned clinical implication in terms of crisis score, it could be used as a biomarker indicative of HU effectiveness.

We could also speculate on the fact that HU could partially restore splenic function favoring IgM memory B-cell output. This is a particular B-cell subset, normally resident in the splenic marginal zone, displaying a wide array of both innate-like and adaptive immune responses. In fact, it is able to differentiate both into IgA-producing plasma cells, through a complex splenic/gut axis, and into IgG-producing switched-memory and plasmacytic B cells, in a germinal center-related T-dependent and T-independent fashion [36,41,42].

No statistically significant difference was found between the SCD and control groups regarding infections; furthermore, no correlations were detected between infection score and immunological parameters. This could depend on an optimal adherence in the SCD group to vaccination against encapsulated bacteria and to chemoprophylactic recommendations, taking into account the low sample size and limited observational time period as potential biases.

This project’s primary limitations resulted from the sample size, its retrospective nature and the different ethnic composition between the SCD and control groups. Another issue worthy of further discussion, but not investigated in our study, regards the assessment in the SCD group of splenic function through Howell–Jolly body or pitted erythrocyte detection in a peripheral blood smear, as well as through an imaging-guided detection of spleen size, radionuclide uptake and major thromboembolic events involving celiac/splenic vessels or through CD19+ IgM memory B-cell count by venous blood sampling [2,3,4,43].

## 5. Conclusions

Our study offers an in-depth immunological characterization of SCD children, taking into account contemporary T- and B-cell subsets, speculating on preliminary data sustaining a plausible clinical implication of baseline and HU-related immunophenotypical alterations in terms of the vaso-occlusive crisis number in SCD patients. Nonetheless, these results need to be validated in larger samples.

These considerations endorse the need for further projects systematically analyzing SCD subjects and other hypo-/a-splenic individuals, in order to better define criteria reliably assessing splenic dysfunction and to unveil the role of the immunological state in the development of hypo-/a-splenism comorbidities, particularly overwhelming post-splenectomy infections and vaso-occlusive crises.

The comprehension of this link could lead to a more appropriate management of hypo-/a-splenic patients, providing a rationale for novel category-specific strategies and patient-tailored approaches in terms of immuno-chemoprophylaxis.

## Figures and Tables

**Table 1:** General characterization of the SCD cohort, HU+/HU− and splenectomized/unsplenectomized groups.

		Total SCD Cohort (*n* = 19)	HU+ Group (*n* = 12)	HU− Group (*n* = 7)	Splenectomized Group (*n* = 3)	Unsplenectomized Group (*n* = 16)
**Categorical Variables**		**No.**	**No.**	**No.**	**No.**	**No.**
Gender	Male	10	06	04	02	08
	Female	09	06	03	01	08
Ethnicity	African	14	07	07	02	12
	Caucasian	05	05	00	01	04
SCD genotype	Hb SS	12	07	05	02	10
	Hb Sβ0-thalassemia	06	05	02	01	06
	Hb SC	01	00	01	00	01
Patients with more than two painful crises per year		/	08	/	02	06
Patients on chemoprophylaxis		7	3	4	0	7
**Continuous Variables**		**Mean**	**Mean**	**Mean**	**Mean**	**Mean**
Age at enrollment in the study (range 3–23) (years)		11	13	07	18	10
Age at the start of hydroxyurea (y)		/	10	/	17	11
Duration of therapy with hydroxyurea (years)		/	04	/	02	05
Dosage of hydroxyurea (mg/kg/die)		21	/	21	21	21
Time between splenectomy and enrollment (years)		/	/	/	12	/

Abbreviations: HU, hydroxyurea; No., number; SCD, sickle cell disease; /, data not available.

**Table 2:** Anagraphic and clinical features of the control group.

		Control Group (*n* = 24)
**Categorical Variables**		**No. (%)**
Gender	Male	13 (54%)
	Female	11 (46%)
Ethnicity	Caucasian	23 (96%)
	Asian	01 (4%)
Cause of referral to hospital	Recurrent URTI	13 (54%)
	Recurrent fever	05 (21%)
	Recurrent tonsillitis	01 (4%)
	EBV virus infection	01 (4%)
	Recurrent herpetic infection	01 (4%)
	Reactive lymphadenopathy	01 (4%)
	Recurrent abdominal pain	01 (4%)
	Transient childhood hypogammaglobulinemia	01 (4%)
**Continuous Variables**		**Mean**
Age at enrollment in the study (range 1–24) (y)		06

Abbreviations: No., number; URTI, upper respiratory tract infection; y, years.

**Table 3:** Comparison of immunologic parameters between the SCD HU−, SCD HU+ and control groups (SE: standard error).

		Control Group (*n* = 24)	SCD HU− Subgroup (*n* = 7)	T-Test (Controls vs. SCD HU− Group)	SCD HU+ Subgroup (*n* = 12)	T-Test (Controls vs. SCD HU+ Group)
Variables		Mean ± SE	Mean ± SE	*p*-Value	Mean ± SE	*p*-Value
*Basic features*	WBC (cell/uL)	8058.72 ± 545.18	12877.14 ± 1688.76	* 0.001	8365.83 ± 1060.16	0.776
	Neutrophils (cell/uL)	3621.40 ± 375.92	6072.15 ± 1251.19	* 0.016	4319.35 ± 700.68	0.342
	Neutrophils (%†)	43.73 ± 2.53	45.89 ± 5.08	0.693	49.84 ± 3.37	0.164
	Lymphocytes (cell/uL)	3657.5 ± 305.48	4615.71 ± 688.74	0.166	3382.00 ± 304.18	0.573
	Lymphocytes (%†)	46.32 ± 2.33	38.51 ± 5.97	0.155	39.94 ± 2.97	0.112
	CD3+ PAN-T cells (%‡)	68.75 ± 1.25	53.28 ± 4.09	* 0.000	67.08 ± 2.90	0.541
	CD4+/CD8+ ratio	1.81 ± 0.11	1.84 ± 0.24	0.917	1.85 ± 0.09	0.818
*CD4+ T-cell* *subsets*	CD3+CD4+ T cells (cell/uL)	1426.17 ± 115.71	1553.85 ± 221.58	0.603	1534.16 ± 223.95	0.637
	CD3+CD4+ T cells (%‡)	39.66 ± 1.29	34.00 ± 1.25	* 0.032	40.16 ± 2.09	0.833
	CD4+CD45RA+CCR7+ naїve T cells (%§)	71.79 ± 1.41	53.43 ± 5.98	* 0.021	63.75 ± 3.40	* 0.045
	CD4+CD45RA−CCR7+ central memory T cells (%§)	19.31 ± 1.13	27.00 ± 1.79	* 0.002	24.16 ± 2.65	0.056
	CD4+CD45RA−CCR7− effector memory T cells (%§)	7.87 ± 0.71	17.57 ± 3.69	* 0.039	10.58 ± 1.33	0.057
	CD4+CD45RA+CCR7− terminal effector memory T cells (%§)	1.08 ± 0.16	2.43 ± 1.15	0.290	1.41 ± 0.43	0.486
	CD4+CD127−CCR7+CD25++ regulatory T cells (%§)	4.16 ± 0.29	3.71 ± 0.36	0.449	3.16 ± 0.42	0.061
*CD8+ T-cell* *subsets*	CD3+CD8+ T cells (cell/uL)	830.62 ± 63.83	900.86 ± 153.12	0.628	751.42 ± 85.10	0.471
	CD3+CD8+ T cells (%‡)	23.04 ± 1.05	20.14 ± 2.35	0.222	22.16 ± 1.42	0.630
	CD8+CD45RA+CCR7+ naїve T cells (%*¶*)	52.33 ± 2.93	39.42 ± 7.17	0.061	53.50 ± 2.54	0.799
	CD8+CD45RA−CCR7+ central memory T cells (%*¶*)	4.20 ± 0.47	4.85 ± 0.94	0.520	4.50 ± 0.73	0.727
	CD8+CD45RA−CCR7− effector memory T cells (%*¶*)	17.25 ± 1.34	19.00 ± 5.81	0.778	16.08 ± 2.33	0.645
	CD8+CD45RA+CCR7− late effector T cells (%*¶*)	26.40 ± 3.22	36.42 ± 5.83	0.148	25.83 ± 2.12	0.883
*Other cell subsets*	CD56+CD16+CD3− natural killer cells (%‡)	11.04 ± 1.03	15.28 ± 1.11	* 0.044	11.00 ± 1.39	0.981
	TCRαβ+CD3+CD4−CD8− double negative T cells (%††)	1.21 ± 0.12	1.42 ± 0.20	0.405	1.18 ± 0.15	0.888
	CD3+γ+δ+ (%‡)	5.41 ± 0.42	4.57 ± 0.72	0.344	4.66 ± 0.59	0.314
*CD19+ B-cell* *subsets*	CD19+ PAN-B cells (cell/uL)	753.00 ± 140.62	1206.28 ± 247.84	0.128	702.33 ± 101.89	0.772
	CD19+ PAN-B cells (%‡)	19.32 ± 1.34	25.17 ± 2.90	0.055	21.23 ± 2.90	0.496
	CD19+IgD+CD27− naïve B cells (%‡‡)	73.50 ± 1.77	86.42 ± 2.20	* 0.001	76.25 ± 7.16	0.715
	CD19+IgM++CD38++ transitional B cells (%‡‡)	5.80 ± 1.02	8.85 ± 1.62	0.156	6.25 ± 0.89	0.781
	CD19+IgD+CD27+ memory B cells (%‡‡)	10.71 ± 0.87	3.21 ± 0.65	* 0.000	10.63 ± 7.05	0.992
	CD19+IgD−CD27+ switched memory B cells (%‡‡)	9.97 ± 0.81	5.41 ± 1.26	* 0.009	6.65 ± 0.79	*0.014
	CD19+CD21+lCD38− CD21low B cells (%‡‡)	3.63 ± 0.29	2.15 ± 0.66	* 0.029	3.27 ± 0.74	0.659
	CD19+IgM−+CD38++ plasmablasts (%‡‡)	1.22 ± 0.24	1.40 ± 0.45	0.728	0.99 ± 0.16	0.440
*Immunoglobulin* *levels*	IgG (mg/dL) §§	910.66 ± 34.28	1233.71 ± 124.63	* 0.001	1596.33 ± 82.02	* 0.000
	IgA (mg/dL) §§	114.5 ± 10.13	147.42 ± 30.15	0.191	233.83 ± 22.64	* 0.000
	IgM (mg/dL) §§	101.83 ± 8.66	79.28 ± 11.08	0.201	86.75 ± 11.67	0.315
	IgE (mg/dL) §§	90.37 ± 24.75	293.71 ± 99.06	0.088	147.58 ± 84.05	0.525

Abbreviations: HU, hydroxyurea; SCD, sickle cell disease; WBC, white blood cells. * Statistically significant. †% total WBC. ‡% total lymphocytes. §% total CD4+ cells. *¶*% total CD8+ cells. ††% TCRαβ+CD3+ cells. ‡‡% total CD19+ cells. §§ SI conversion factor: To convert IgG/IgA/IgM to g/L, multiply values by 10^2^.

## Data Availability

The data supporting the findings of this study are available in the figure and tables of this article.

## Supplementary Materials

The following supporting information can be downloaded at: https://www.mdpi.com/article/10.3390/jcm11113037/s1, Supplementary Table S1: Correlation analysis between mean count of immunological variables and crisis score in SCD group.
