# Supplementary material for: In-Depth Immunological Typization of Children with Sickle Cell Disease: A Preliminary Insight into Its Plausible Correlation with Clinical Course and Hydroxyurea Therapy
Source: J Clin Med. 2022 May 27;11(11):3037. doi: 10.3390/jcm11113037 (PMC9181704; doi:10.3390/jcm11113037)
Supplement: Supplementary file 1 [file jcm-11-03037-s001.zip › jcm-1688505-supplementary.pdf]

Supplementary Table S1. Correlation analysis between mean count of immunological variables and crisis score in SCD group

SCD group		Univariate analysis	Multivariate analysis
Variables		Pearson's R	Pearson's R
Basic features	WBC (cell/ul)	>0.05	>0.05
	Neutrophils (cell/ul)	>0.05	>0.05
	Neutrophils (%†)	>0.05	>0.05
	Lymphocytes (cell/ul)	>0.05	>0.05
	Lymphocytes (%†)	>0.05	>0.05
	CD3+ PAN-T cells (%‡)	>0.05	>0.05
	CD4+/CD8+ ratio	>0.05	>0.05
CD4+ T-cell subsets	CD3+CD4+ T cells (cell/ul)	>0.05	>0.05
	CD3+CD4+ T cells (%‡)	>0.05	>0.05
	CD4+CD45RA+CCR7+ naïve T cells (%§)	>0.05	>0.05
	CD4+CD45RA-CCR7+ central memory T cells (%§)	>0.05	*0.039
	CD4+CD45RA-CCR7- effector memory T cells (%§)	>0.05	>0.05
	CD4+CD45RA+CCR7- terminal effector memory T cells (%§)	>0.05	>0.05
	CD4+CD127-CCR7+CD25++ regulatory T cells (%§)	>0.05	>0.05
CD8+ T-cell subsets	CD3+CD8+ T cells (cell/ul)	>0.05	>0.05
	CD3+CD8+ T cells (%‡)	>0.05	>0.05
	CD8+CD45RA+CCR7+ naïve T cells (%¶)	>0.05	>0.05
	CD8+CD45RA-CCR7+ central memory T cells (%¶)	>0.05	>0.05
	CD8+CD45RA-CCR7- effector memory T cells (%¶)	>0.05	>0.05
	CD8+CD45RA+CCR7- late effector T cells (%¶)	>0.05	>0.05
Other cell subsets	CD56+CD16+CD3- natural killer cells (%‡)	>0.05	>0.05
	TCRαβ+CD3+CD4-CD8- double negative T cells (%††)	>0.05	>0.05
	CD3+γ+δ+ (%‡)	>0.05	>0.05
CD19+ B-cell subsets	CD19+ PAN-B cells (cell/ul)	>0.05	>0.05
	CD19+ PAN-B cells (%‡)	>0.05	>0.05
	CD19+IgD+CD27- naïve B cells (%‡‡)	>0.05	>0.05
	CD19+IgM++CD38++ transitional B cells (%‡‡)	>0.05	>0.05
	CD19+IgD+CD27+ memory B cells (%‡‡)	*0.010	>0.05
	CD19+IgD-CD27+ switched memory B cells (%‡‡)	>0.05	>0.05
	CD19+CD21+lCD38- CD21low B cells (%‡‡)	>0.05	>0.05
	CD19+IgM-+CD38++ plasmablasts (%‡‡)	>0.05	>0.05
Immunoglobulin levels	IgG (mg/dl)§§	>0.05	>0.05
	IgA (mg/dl)§§	>0.05	>0.05
	IgM (mg/dl)§§	>0.05	>0.05
	IgE (mg/dl)§§	>0.05	>0.05

Supplementary Table. *Abbreviations: HU, hydroxyurea; SCD, Sickle cell disease; WBC, white blood cells.*

** Statistically significant.*

† % total WBC.

‡ % total lymphocytes.

§ % total CD4+ cells.

¶ % total CD8+ cells.

†† % TCRαβ+CD3+ cells.

‡‡ % total CD19+ cells

§§ SI conversion factor: To convert IgG/IgA/IgM to g/L, multiply values by 10^2.
